# Association between environmental chemicals co-exposure and peripheral blood immune-inflammatory indicators

**DOI:** 10.3389/fpubh.2022.980987

**Published:** 2022-11-22

**Authors:** Yong Liu, Zhihui Zhang, Dongran Han, Yiding Zhao, Xiaoning Yan, Shengnan Cui

**Affiliations:** ^1^Department of Dermatology, Shaanxi Provincial Hospital of Traditional Chinese Medicine, Xi'an, China; ^2^School of Life Science, Beijing University of Chinese Medicine, Beijing, China; ^3^Department of Plastic Surgery, The First Affiliated Hospital of Xinjiang Medical University, Xinjiang Medical University, Ürümqi, China; ^4^Xiyuan Hospital, China Academy of Chinese Medical Sciences, Beijing, China; ^5^Graduate School, China Academy of Chinese Medical Sciences, Beijing, China

**Keywords:** chemical mixtures, inflammation, peripheral blood, co-exposure, environmental pollution

## Abstract

Chronic inflammation is closely related to chronic inflammatory diseases, autoimmune diseases and cancer. Few studies have evaluated the effects of exposure to multiple chemical combinations on immunoinflammatory related indicators and their possible molecular mechanisms. This study explored the effect of exposure to various chemicals on immune-inflammatory biomarkers and its molecular mechanism. Using data from 1,723 participants in the National Health and Nutrition Examination Survey (NHANES, 2011–2012), the aim was to determine the association between chemical mixtures and immunoinflammatory biomarkers [including White blood cell (Wbc), neutrophil (Neu), lymphocytes (Lym), and Neutrophil-to-lymphocyte ratio (NLR)] using linear regression model, weighted quantile sum regression (WQSR) model, and bayesian nuclear machine regression (BKMR) model. Meanwhile, functional enrichment analysis and protein–protein interaction network establishment were performed to explore the molecular mechanism of inflammation induced by high-weight chemicals. In the linear regression model established for each single chemical, the four immunoinflammatory biomarkers were positively correlated with polycyclic aromatic hydrocarbons (PAHs), negatively correlated with perfluoroalkyl substances (PFASs), and positively or negatively correlated with metallic and non-metallic elements. WQSR model showed that cadmium (Cd), perfluorooctane sulfonic acid (PFOS) and perfluorodecanoic acid (PFDE) had the highest weights. In BKMR analysis, the overall effect of chemical mixtures was significantly associated with Lym and showed an increasing trend. The hub genes in high-weight chemicals inflammation-related genes were interleukin-6 (IL6), tumor necrosis factor (TNF), and interleukin-1B (IL1B), etc. They were mainly enriched in inflammatory response, Cytokine-cytokine receptor interaction, Th17 cell differentiation and IL-17 signaling pathway. The above results show that exposure to environmental chemical cocktails primarily promotes an increase in Lym across the immune-inflammatory spectrum. The mechanism leading to the inflammatory response may be related to the activation of IL-6 amplifier by the co-exposure of environmental chemicals.

## Introduction

Immune system is a disease defense system composed of a series of biological structures and processes in organisms. Leukocyte is a part of immune system, which refers to the general name of cells that can produce specific immune response. Neutrophils and lymphocytes account for 60–70% and 18–42% of the total number of leukocytes, respectively. They are responsible for immune surveillance, immune defense and immune homeostasis *in vivo*, and their quantity and quality are closely related to human health (1, 2). Neutrophils are associated with acute injury and repair, chronic inflammatory process, cancer and autoimmunity. Lymphocytes mediate cellular immunity, humoral immunity and killing effect on tumor cells and virus-infected cells (3). The neutrophil–lymphocyte ratio (NLR) is a cost-effective biomarker reflecting the balance between systemic inflammation and immunity. The quantitative evidence presented suggests an association between NLR and poor outcomes in patients across a wide spectrum of diagnoses (such as coronary heart disease, psoriasis, stroke, diabetes, obesity, metabolic syndrome, psychiatric diagnosis, cancer of solid organs, anemia, stress, etc.), stages of disease and courses of treatment (4–10).

More and more convincing evidence shows that chemical exposure in the environment is related to the increasing incidence of many chronic diseases, including tumors. It is reported that perfluoroalkyl substances (PFASs) exposure can increase the risk of some cancers/malignancies, and may also lead to elevated cholesterol levels, changes in liver enzymes, decreased vaccine response, cardiovascular diseases, immune-mediated diseases, increased risk of hypertension or preeclampsia in pregnant women, and a slight decrease in the birth weight of babies (11). Long-term exposure to polycyclic aromatic hydrocarbons (PAHs) can mediate adverse effects on immune system, which is teratogenic, genotoxicity, and carcinogenic. It is related to cardiovascular diseases, diabetes, liver and kidney damage, etc., and is also a risk factor for early childhood wheezing, multiple sclerosis and stroke (12, 13). Metal exposure occurs in a chronic way, leading to a pathogenic immune response that lasts for months or years. Therefore, chronic inflammatory reaction, allergic and autoimmune diseases have been reported to be affected by environmental metal exposure (14).

In a word, the above three kinds of environmental pollutants are exposed to people almost every day, and they are related to many chronic diseases, such as tumor and immune diseases. Wbc, Neu, Lym, and NLR are very important biomarkers related to immune inflammation. The impact of these environmental pollutants on the three immune-inflammatory biomarkers may be a potential mechanism for the high incidence of various diseases. To our knowledge, there are few large-scale epidemiological studies on the effects of co-exposure of PFASs, PAHs, metallic and nonmetallic elements on immune-inflammatory biomarkers. In the present research, a cross-sectional study was conducted based on population data from the NHANES (2011–2012). The impact of single chemical contaminant was analyzed using multiple logistic regression. Furthermore, weighted quantile sum regression (WQSR) and Bayesian kernel machine regression (BKMR) models were applied to investigate the roles of chemicals co-exposure (PFASs, PAHs, metallic and non-metallic elements) in peripheral blood immune-inflammatory indicators. The results provided novel epidemiological evidence on the associations of chemicals co-exposure with immune inflammatory risk, and contributed to the identification of the hazardous factors of immune inflammatory reaction.

## Materials and methods

### Study population

**Figure 1:**
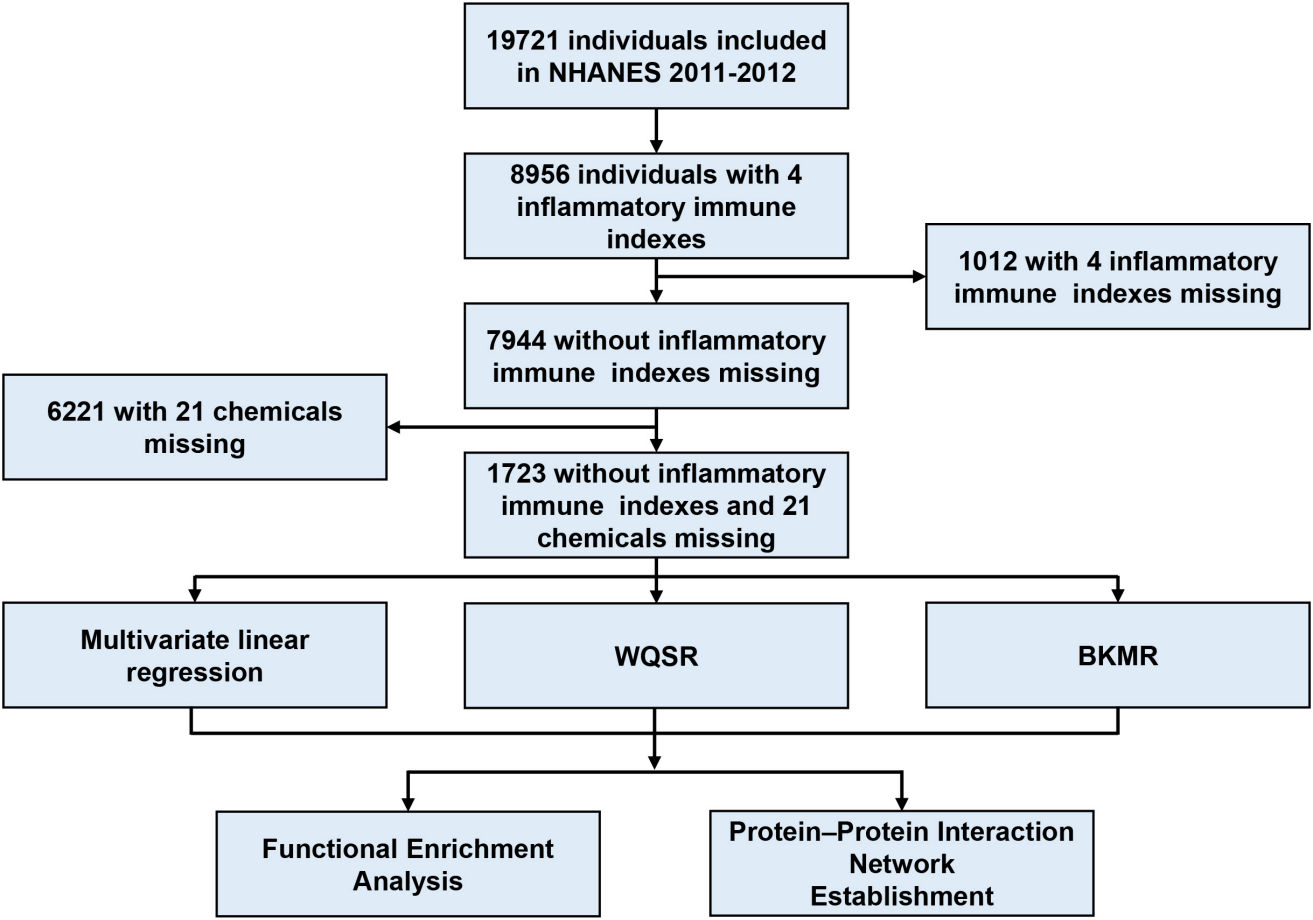
Flowchart for inclusion of study participants.

The National Health and Nutrition Examination Survey (NHANES) is a cross-sectional study that aims to assess the health and nutrition status of the American population by collecting demographic, dietary, inspection and laboratory data. NHANES survey was approved by the Ethics Review Committee of the National Health Statistics Research Center. We have integrated the data of six cycles from 2005 to 2016, and found that only the data of this cycle from 2011 to 2012 is complete and contains the measured values of Wbc, Neu, Lym, PFASs, PAHs, metallic and non-metallic elements. Then we further integrated the data of this cycle from 2011 to 2012. Finally, the basic demographic information and the measurement data of Wbc, Neu, Lym, PFASs, PAHs, metallic and non-metallic elements of 1,723 subjects were complete and had no missing values, which can be used as the final included data of this study. The flow chart is presented in Figure 1.

### Measurement of environmental chemicals and outcomes

Blood and urine samples were collected at the Mobile Testing Center (MECs) and stored temporarily at −20 °C until analyzed by the Laboratory Science Division, Organic Analytical Toxicology Division, National Center for Environmental Health (Atlanta, GA). Complete information on chemical measurement methods is available in the NHANES laboratory method. In this study, we included 21 environmental chemicals (including 7 PFASs, 10 PAHs, and 4 metallic and non-metallic elements) with potential effects on immunoinflammatory biomarkers (Supplementary Table 1). According to the NHANES Analysis Standard, the limit of detection (LOD) divided by the square root of two was used to replace the values below the LOD. In addition, natural logarithm transformation (LN transformation) is performed on the concentration of environmental chemicals to improve the normal distribution because of their right-skewed distribution.

### Covariate

The potential covariates were identified in this study based on subjective knowledge, and a literature review (15–18). The full model was then built using these variables. Covariates including age, gender, ethnicity, education levels, income and body mass index (BMI) were obtained by direct interview. Age was treated as continuous variables. The categories of other covariates were as follows: gender (female, male), ethnicity (White, Black, Mexican, other), education levels (<6th grade, 6–9th grade, 9–12th grade, College graduate or above, Some college or AA degree), BMI (<25 kg/m^2^, 25 kg/m^2^ < 30 kg/m^2^, and 30 kg/m^2^), income (0–20,000$, 20,000–55,000$, 55,000–100,000$, and >100,000$).

### Statistical analysis

#### Correlation among chemicals and immune inflammation indicators

We used the measured values of co-exposed chemicals and immune-inflammatory markers to conduct Pearson correlation analysis within and between groups to study the correlation strength among 21 chemicals and 4 immune inflammation indicators. The R package “ggcorrplot (version 0.1.3)” was used to calculate the correlation coefficient with a *P*-value < 0.01 being regarded as valid correlations.

#### Multivariate linear regression

First, geometric mean (GM), arithmetic mean (AM) and interquartile ranges (IQR) were calculated for each exposure and immune inflammation index. Then, we fitted the multivariate linear regression to evaluate the effect of a single chemical on immune inflammation indicators by comparing Q2, Q3, Q4 to Q1. We estimated the effects of exposures on immune inflammation indicators of each quantile compared with the reference β (the coefficient of the multivariate linear model). We also calculated the *P for trend* by fitting the level-converted chemical concentration (ln-transformed) into our linear model to ensure the dose-response evidence of our results. We adjusted all the multiple linear models based on age, gender, ethnicity, education levels and BMI.

#### WQSR model

The WQSR, a supervised approach, was used to evaluate the impact of environmental mixtures and identify the predominant exposure (19). As part of the procedure, the study sample was randomly divided into a validation dataset (60%) and a training dataset (40%). In the training dataset, every chemical was first scored into quartiles. After that, a total quantile score was created for each individual by summing the quartiles. The empirical weights of each chemical in the mixture were estimated through Bootstrapping using the training data set. Then these weights were used to create WQSR scores representing the whole mixture, and their statistical significance was tested in the validation dataset (20). Chemicals with an estimated weight > 0.048 (1/21) were considered to have a significant contribution to WQSR score (20). As the WQSR method was based on the assumption that all mixed chemicals have the same effect on the outcome, we evaluated the positive and negative WQSR scores, respectively. The weight of WQSR score was estimated using 1,000 bootstrap samples from the training data set (40%), and the statistical significance of WQSR score of immune-inflammatory spectrum was tested in the validation data set (60%) (19). WQSR is implemented with R package“gWQS” (version 3.0.4).

#### BKMR model

BKMR is a supervised method to identify the uncertain exposure-outcome relationship by non-parametric method (kernel function) and then evaluate the exposure mixtures. BKMR can model the exposure-response function flexibly without prior explanation (21). Considering the high correlation between similar chemicals and the non-additive and non-linear relationship between chemicals, this method not only parametrically models the additional covariates of interest, but also estimates the potential interaction and non-linear correlation. The equation expression of BKMR model is as follows:


$$\begin{array}{l} Yi=h(PFDEi,PFOSi,PFUAi,PFNAi,PFOAi,PFHSi,MPAHi,\\ P01i\ldots )+\beta qZi+ei \end{array}$$

The function *h* in the equation is an exposure-reaction function that can take into account the non-linearity and/or interaction among various chemical components in the mixture, and Z = Z1, Z2 …, Z*q* indicates that there are *q* potential confounding factors, *Zi*, and β represented covariates and their coefficients, respectively. In this study, Gaussian kernel function with component variable selection strategy was used, and all chemicals were standardized and ln transformed. After 20,000 iterations of fitting the final model with Markov Chain Monte Carlo (MCMC) sampler, the posterior inclusion probabilities (PIPs) of each chemical was calculated, and the estimated value of exposure-outcome function was generated. The PIP threshold of 0.5 is usually used to judge whether this chemical is important (22). BKMR is helpful to understand the health consequences of single exposure and their interactions. In order to evaluate the overall effect of chemical combination on immune inflammatory cell indicators, we first compared the results when all chemicals were set at 25th, 30th, 35th, 40th, 45th, 55th, 60th, 65th, 70^th^, or 75th percentile with those when all chemicals were set at 50th percentile. Second, interactions between chemicals can also be assessed by estimating the change in outcome level associated with a change in individual chemical concentrations, at varying levels (e.g. 25th, 50th, 75th percentile) of one or more additional chemical (20). R software package “bkmr” (version 0.2.0) was used for analysis.

#### Functional enrichment analysis and protein–protein interaction network

##### Establishment

In the present study, the chemicals - genes associated with inflammation were obtained from CTD (http://CTD.mdibl.org), which is a robust, publicly available database that provides manually curated information about chemical–gene/protein interactions. In order to explore chemical-immune inflammation genes function, pathway and disease enrichment characteristics, Gene ontology (GO) and Kyoto Encyclopedia of Genes and Genomes (KEGG) pathway and DisGeNET (DGN) enrichment analyses were evaluated using R packages “clusterProfiler (version 4.2.2) ” and “DOSE (version 3.20.1)” (23). In order to further discover and explore the chemical-immune inflammation genes interaction at the protein level, chemical-immune inflammation genes were uploaded to the Search Tool for the Retrieval of Interacting Genes (STRING) database (Version 10.0, http://string-db.org) and a combined score > 0.4 (medium confidence score) was considered significant. Cytoscape software (Version 3.4.0, http://www.cytoscape.org/) was then used to construct a protein-protein interaction (PPI) network (24).

## Results

### Characteristics of eligible subjects

A total of 1,723 participants were included in this study, including 879 males, accounting for 51.02% of the total number of participants, and 844 females, accounting for 48.98%. Other demographic details (for example, age and race), socioeconomic status (for example, education level and annual household income), and Body Mass Index are shown in Table 1.

**Table 1:** Characteristics of study population (*n* = 1,723).

**Covariate**		***N* (%)**
Gender	Male	879 (48.98)
	Female	844 (51.02)
Age (years)	12–24	470 (27.28)
	25–40	399 (23.16)
	41–59	434 (25.19)
	>60	420 (24.38)
Race/ethnicity	White	606 (35.17)
	Black	446 (25.89)
	Mexican	193 (11.20)
	Other	478 (27.74)
Body mass index	< 25 kg/m^2^	649 (37.67)
	25 kg/m^2^ < 30 kg/m^2^	513 (29.78)
	30 kg/m^2^	561 (32.56)
Income	0–20,000$	397 (23.04)
	20,000–55,000$	701 (40.69)
	55,000–100,000$	328 (19.04)
	>100,000$	297 (17.24)
Education	< 6th grade	14 (0.81)
	6–9th grade	297 (17.24)
	9–12th grade	593 (24.42)
	College graduate or above	393 (22.81)
	Some college or AA degree	426 (24.72)

### Associations between environmental chemicals and immunoinflammatory

#### Biomarkers

Supplementary Table 1 describes the distribution of 21 chemical exposures and 4 peripheral blood immune-inflammatory biomarkers. The detection rate of PFASs was 87.5%, PAHs was over 95%, As was 96.5%, the other three metallic and nonmetallic elements were 88.4%, and the detection rate of peripheral blood immune inflammatory cells was 88.4%. Supplementary Figure 1 displays the Pearson correlation coefficients (Corr) among 21 chemicals and 4 immune inflammation indicators (*P*-value < 0.01, Corr ranging from −0.16 to 0.95). The same kind of chemicals tend to have strong correlation, and most of them are positively correlated with each other. For example, there is a strong correlation between P04 and P03 (Corr = 0.95). The correlation between different kinds of chemicals is weak and mostly negative, for example, there is a weak negative correlation between P02 and PFUA (Corr = −0.15). Environmental chemicals clustered and altered in a categorical way, indicating that a highly coordinated exposome regulatory network underlies the mechanism of immune- inflammatory changes in human body.

### Generalized linear regression model to assess the association between environmental chemicals and immunoinflammatory biomarkers

We used multiple linear regression to assess the association of each chemical with immunoinflammatory biomarkers (Supplementary Table 2). In multiple linear regression analysis, after adjusting for all covariates, Wbc was negatively correlated with PFDE and PFUA in PFASs, positively correlated with all PAHs, negatively correlated with As, and positively correlated with Cd and Hg compared to the first quartile exposure level. Neu was negatively correlated with PFDE, PFOS, PFUA and PFNA in PFASs, positively correlated with all PAHs, negatively correlated with As and Hg, and positively correlated with Cd. Lym was positively correlated only with PFNA, P01, P02, P03, P04, As, and Cd. NLR was negatively correlated with PFDE, PFOS, PFUA, PFNA and PFOA, positively correlated with P02, P03 and P04, P05, P07, P10, P17, and P19, and negatively correlated with Hg. Overall, we found that four immunoinflammatory biomarkers were positively correlated with PAHs, negatively correlated with PFASs, and positively or negatively correlated with metal or non-metal.

### WQSR model to assess the association of environmental chemicals co-exposure with immunoinflammatory biomarkers

**Figure 2:**
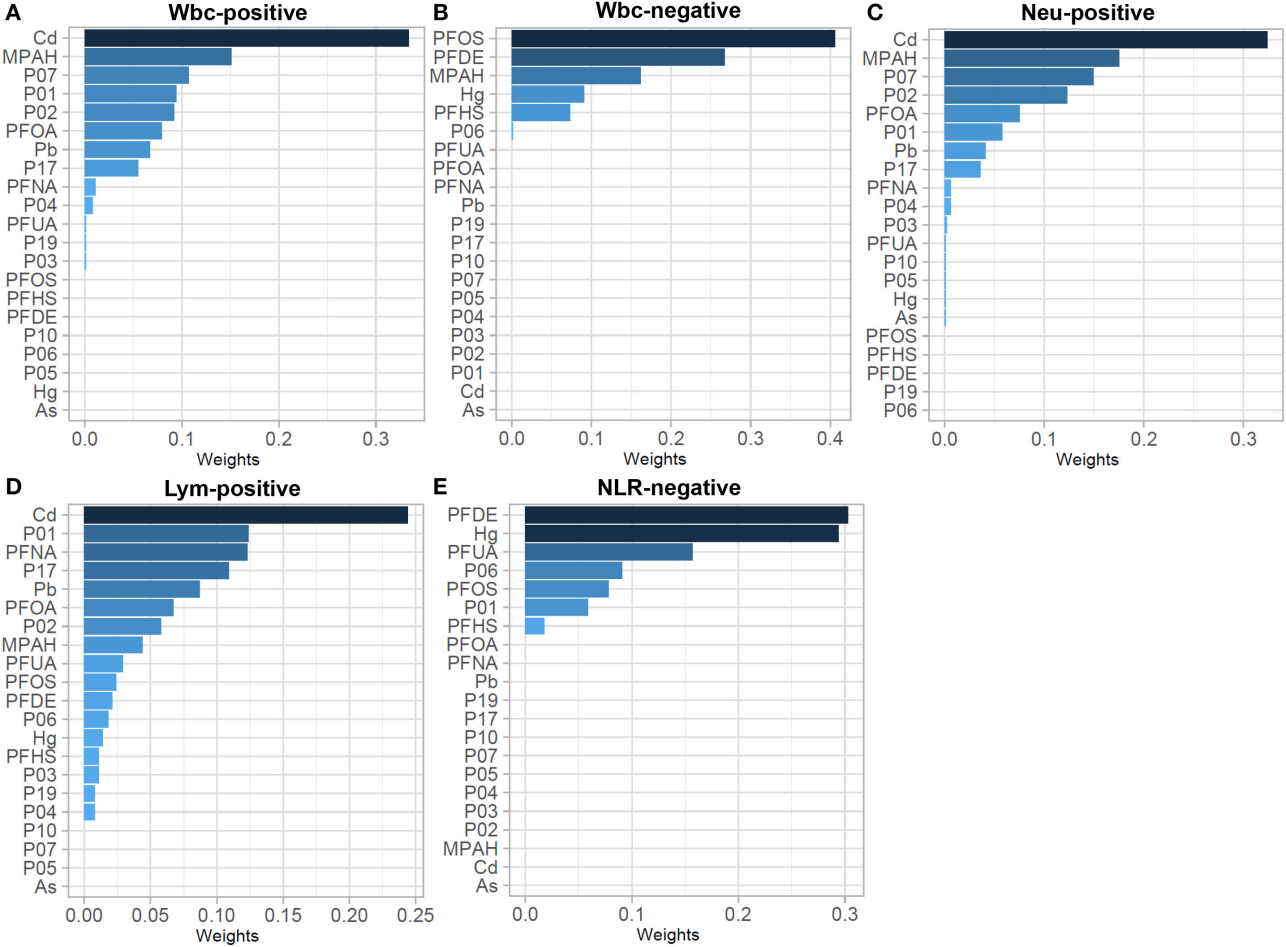
WQSR weights in the WQSR model between immunoinflammatory biomarkers and WQSR index of total 21 environmental chemical mixtures. **(A)** Weights in WQSR index in the models of Wbc-positive. **(B)** Weights in WQSR index in the models of Wbc-negative. **(C)** Weights in WQSR index in the models of Neu-positive. **(D)** Weights in WQSR index in the models of Lym-positive. **(E)** Weights in WQSR index in the models of NLR-negative.

To analyze changes in the immune-inflammatory indicators elicited by mixed exposures, we fitted a WQSR model to assess the effects of exposure to 21 environmental chemicals on immune-inflammatory indices (Supplementary Table 3). Among the positive correlations, total exposure to environmental chemicals was positively correlated with Wbc, Neu and Lym with Cd, MPAH, and P07 had greater effects on Wbc and Neu, and Cd, P01, and PFNA were relatively high-weight chemicals in the Lym model. In the negative correlation, total environmental chemical exposure was significantly negatively correlated with immune-inflammatory indices (Wbc, NLR), with PFOS and PFDE having a greater effect on Wbc, and PFDE and Hg having a greater effect on NLR (Figure 2).

### The relationship between chemical levels and immunoinflammatory

#### Biomarkers using the BKMR model

We used the BKMR approach to further identify the impacts of chemical mixtures, taking into account the previous study's limits of linearity and interactions. Supplementary Table 4 summarizes the PIPs determined by the BKMR model for the chemical. PIPs were used as a variable importance measure, with higher values (closer to 1) indicating greater significance. PFUA had the highest PIPs of the total chemicals in models of Wbc, Neu and NLR, with a downward trend, while PFHS had the highest PIPs in Lym model, with an upward trend, and the total chemicals were fixed at 25th, 50th, and 75th percentile (Supplementary Figure 2).

**Figure 3:**
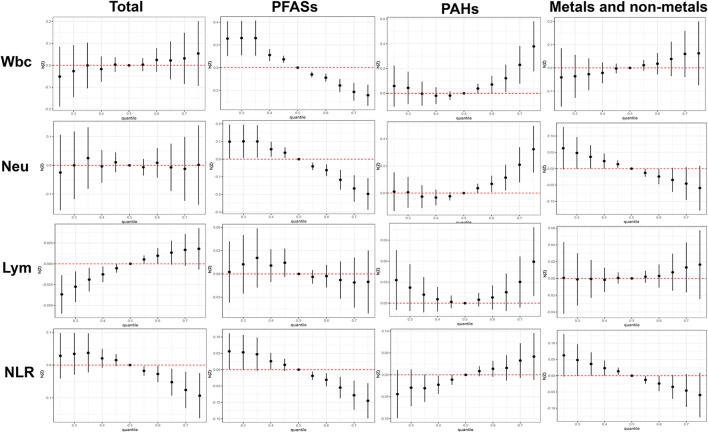
Combined effects of the total chemical mixtures and different subgroups (PFASs, PAHs, Metals and non-metals) on Wbc, Neu, Lym, and NLR when all the chemicals were held at particular percentiles compared to their medians. The results were assessed by the BKMR model and adjusted for age, gender, ethnicity, education levels, BMI.

The total effect of the different kinds of chemical mixtures (total chemical mixtures, PFASs, PAHs and Metals) on immunoinflammatory biomarkers is shown in Figure 3. When total chemical mixtures were at above the 60th percentile, compared to the 50th percentile, Wbc and Lym increased significantly, while NLR decreased significantly, Neu has no clear trend.

We have also investigated the univariate (single chemical) exposure response functions of chemical exposure on immunoinflammatory biomarkers (Supplementary Figure 3). The results indicated that PFAHs contained negative associations with the 4 immunoinflammatory biomarkers, while PAHs exhibited inverse relationship. Metallic and non-metallic elements have positive and negative trends.

### Functional enrichment analysis and PPI network establishment of high-weight chemicals inflammation-related genes

Table 2 shows the results of immune inflammation-related targets of 21 chemicals obtained through the analysis of 3 models. Finally, 9 high-weight chemicals including PFDE, PFUA, PFOS, As, Hg, P01, P02, P04, and Cd were obtained. From CTD data, 102 genes related to inflammation of high-weight chemicals were obtained (Supplementary Table 5).

**Table 2:** A summary of results using the three approaches to evaluate the link between 21 chemicals and immunoinflammatory biomarkers (*n* = 1,723).

**Approaches**	**Indicators**	**Wbc**	**Neu**	**Lym**	**NLR**
Linear regression model (chemicals as continuous variables)	Strong negative associations (lightest β indicators)	PFDE, PFUA, As	PFDE, PFUA, PFOS	As	PFDE, Hg, PFUA
	Strong positive associations (highest β indicators)	P02, P04, P03	P02, P04, P03	P01, P02, P03	P02, P04, P01
QWSR model	Highest negative weights	PFOS, PFDE, MPAH	Not significant	Not significant	PFDE, Hg, PFUA
	Highest positive weights	Cd, MPAH, P07	Cd, MPAH, P07	Cd, P01, PFNA	Not significant
BKMR model	Negative trend (highest PIPs)	PFUA, As, PFDE	PFUA, As, PFDE	Not significant	PFUA, P01, P17
	Positive trend (highest PIPs)	P04, P03, P01	P04, P03, P02	PFHS, As, PFOA	PFDE, Hg, PFOS

**Figure 4:**
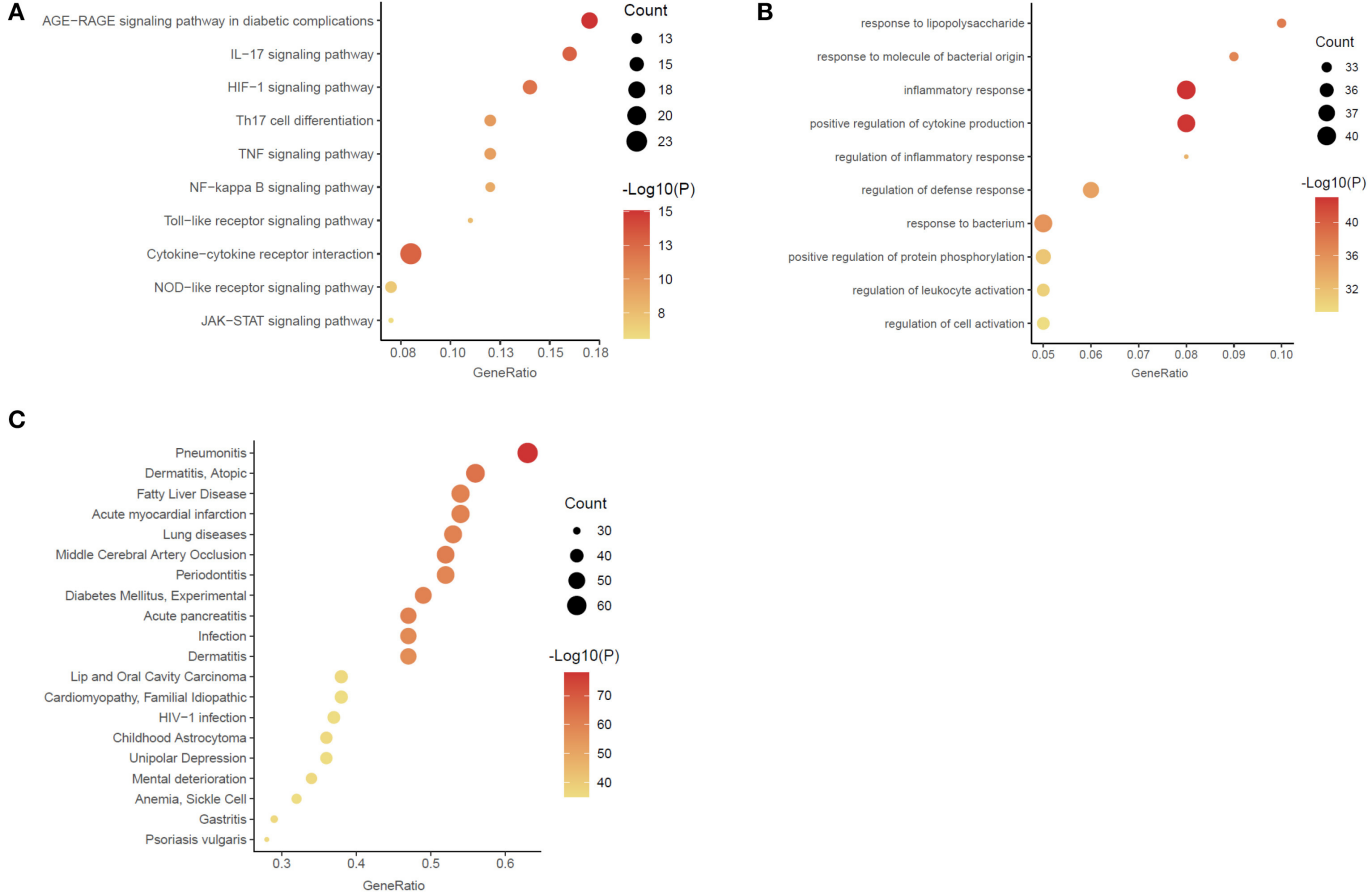
Enrichment analysis of the chemicals – inflammation genes **(A)** Gene ontology (GO) functional enrichment analysis. **(B)** Kyoto Encyclopedia of Genes and Genomes (KEGG) enrichment analysis. **(C)** DisGeNET (DGN) enrichment analysis.

Through enrichment analysis of high-weight chemicals inflammation-related genes, we learned the relationship between these genes and functions, pathways, and diseases. These genes were enriched in the function of inflammatory response and positive regulation of cytokine production (Figure 4A). The main pathways were the Cytokine-cytokine receptor interaction, Th17 cell differentiation and the IL-17 signaling pathway (Figure 4B). The diseases were mainly enriched in Pneumonitis, Atopic dermatitis, Gastritis, and Middle Cerebral Artery Occlusion (Figure 4C).

**Figure 5:**
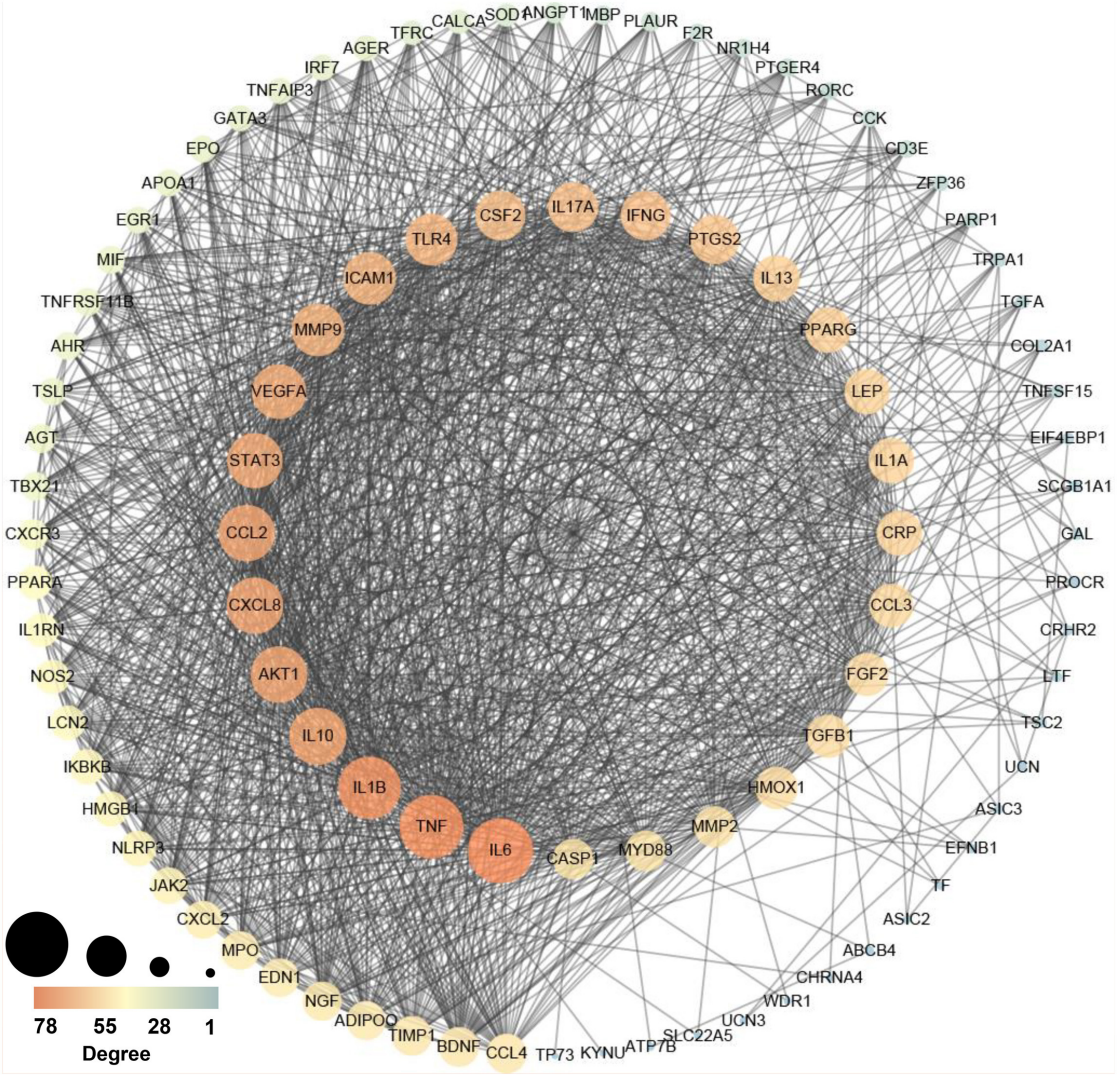
Protein–protein interaction (PPI) network establishment of the chemicals – inflammation genes (A larger value leads to a larger size, and a larger value leads to an orange color).

To elucidate the interaction between high-weight chemicals inflammation-related genes, Cytoscape visualized the STRING-based PPI network for inflammation-related genes of high-weight chemicals. By analyzing the 102 genes, we got a network interaction graph with 96 nodes and 1,387 edges, where nodes represented genes, edges represented connections between two genes, and degree value represented the strength of association between genes (Figure 5). More precisely, the top 10 hub genes of inflammation-related genes of high-weight chemicals were IL6, TNF, IL1B, IL10, AKT1, CXCL8, CCL2, STAT3, VEGFA, and MMP9 (Figure 5).

## Discussion

In this study, single and multiple exposure models (BKMR model and WQSR model) were integrated to evaluate the immune-inflammatory response caused by exposure of mixed chemicals, and the relationship between immune-inflammatory indicators and co-exposure of environmental chemicals was comprehensively explored. Using three statistical models, the study determined the effect of chemical combinations on immunoinflammatory biomarkers. To our knowledge, this is the first study to examine the association between chemical exposure and parameters related to peripheral blood immune inflammation. On the one hand, generalized linear regression showed that three kinds of chemicals (PFASs, PAHs and Metals) were related to peripheral blood immune inflammation related indexes. In the positive WQSR model, WQSR showed that Cd had the greatest influence on Wbc, Neu and Lym, while in the negative WQSR model, WQSR showed that PFOS and PFDE had the largest weights of Wbc and NLR, respectively. On the other hand, in the BKMR model, we found that the univariate exposure response function was approximately correlated with the single exposure model, and mixed exposure was significantly positively correlated with Lym and significantly negatively correlated with NLR. Although mixed exposure was not significant with Wbc, there was an increasing trend, while there was no obvious monotonic trend with Neu.

It is well known that generalized linear regression models are commonly used to assess the health effects of chemicals when they are limited to one chemical or a group of similar chemicals, which is simple and easy to understand. In this study, we found high correlations among the same chemicals. Therefore, when the research object is non-linear interaction, multicollinearity, exposure to multiple chemicals, or there is a high correlation between chemicals, the generalized linear regression model is not applicable, and the positive or negative correlation results between chemicals and immune inflammation related indicators will be distorted due to the mixed influence of other chemicals (25).

People seldom come into contact with a single chemical substance in the real environment, but are generally affected by a variety of chemical substances, and there are interactions and non-linear interrelations among them. Therefore, it is necessary to explore the impact of mixed exposure on human health based on the two new methods of WQSR and BKMR. When WQSR is used to study the co-exposure effect of chemical mixtures, this method can only evaluate the co-exposure effect of chemicals in a single effect direction, but it has limitations in evaluating the interaction between chemicals and the double effect direction (19). BKMR is a supervised method for evaluating exposure mixtures, which characterizes the co-exposure effects under different effect directions by non-parametric methods (especially kernel functions). The method can also evaluate potential interactions and non-linear relationships. However, BKMR has limitations when estimating involves simultaneous exposure to high and low levels of chemicals (21). This study integrates various statistical methods to evaluate the effects of chemical mixture co-exposures on the immune-inflammation indicators, demonstrating that mixed chemical exposures (PFAHs, PAHs, Metallic and non-metallic elements) can indeed affect the immune-inflammation indicators, which in turn affects health.

In this study, we found different trends in the influence of different chemicals on immune-inflammatory indicators. Therefore, in the case of co-exposure to multiple chemicals, the final influence trend of immune-inflammatory indicators may be influenced by exposure measurement of different chemicals, and the higher exposure measurement has a higher weight on the overall trend. In this study, we found that PFDE, PFUA, As and Hg in the comprehensive exposure chemicals had a high negative weight on the immune inflammation spectrum, while P01, P02, P04, and Cd had a high positive weight on the immune inflammation spectrum.

PFASs belong to long-chain perfluoroalkyl carboxylic acids (PFDE and PFUA, etc.) with significant persistence and bioaccumulation potential (26), which have been widely used in food packaging, household cleaning products, furniture, interior decoration, textiles, cosmetics, medical equipment, and other fields (27–29). Polycyclic aromatic hydrocarbons (PAHs) are a group of chemicals formed during the incomplete combustion of coal, oil, gas, and garbage including vehicle exhaust, bitumen, coal tar, wildfires, agricultural burning, etc., (13, 30). Arsenic (As) is a toxic metal that is widely distributed in the environment and is present in soil, food, and water, leading to unavoidable human exposure to arsenic. Cadmium is mainly released from nickel-cadmium batteries, coatings and coatings, plastic stabilizers, fossil fuel combustion, phosphate fertilizer and garbage incineration. Mercury pollution mainly comes from coal burning, non-ferrous metal production and cement production (31).

Long-term abnormal action of immune-inflammatory cells in the body can lead to IID (immune-inflammatory disease) (32). One study noted an increased risk of myocardial infarction (MI) with IIDs, 69% increased risk of RA, 41% increased risk of psoriatic arthritis, and increased cardiovascular risk was also observed in patients with psoriasis (33, 34). A recent study showed that the presence of one IID increased the risk of patients developing additional IIDs by 5–62%, and developing any two secondary IIDs increases the risk by 3–75% (35).

This study found that when exposed to all chemicals (PFAHs, PAHs, Metallic and non-metallic elements), Wbc and Lym showed an increasing trend, NLR showed a decreasing trend, while Neu showed no obvious monotonic trend. Since neutrophils and lymphocytes are the main components of white blood cells, NLR reflects the balance between the two aspects of the immune system: acute and chronic inflammation (indicated by neutrophil counts) and adaptive immunity (lymphocyte counts) (6, 36). Therefore, lymphocyte immunity is mainly activated under comprehensive chemical exposure. Lymphocytes are composed of T lymphocytes, B lymphocytes and NK cells, among which T lymphocytes are involved in cellular immunity and are also considered to be major drivers of many inflammatory and autoimmune diseases (37).

Environmental pollutants (such as PAHs and PFASs) can enter human body through respiratory tract, digestive tract and skin, and interact with immune cells to enhance the adaptive immune response of type 2 helper T lymphocytes (Th2) and type 17 helper T lymphocytes (Th17) (38). Aromatic hydrocarbon receptor (AhR) is a cytoplasmic environment sensing receptor. High expression of AhR in Th17 cell can also promote Th17 cell differentiation and is capable of crosstalk with various inflammatory and antioxidant transcription factors, such as RORγ T, STAT1, Nrf2 and NFκB (39). Dysregulation of Th17 cell immunity can induce a variety of immune- inflammatory diseases, such as asthma and chronic obstructive pulmonary disease (COPD), periodontitis, rheumatoid arthritis, systemic lupus erythematosus, psoriasis, systemic sclerosis, and inflammatory bowel disease, etc., (40).

Inflammation is a complex process in which various cells, such as B lymphocytes, T lymphocytes, epithelial cells, etc.), pro-inflammatory cytokines [such as IL-1β, IL-6 and tumor necrosis factor α (TNFα)], transcription factor nuclear factor κB (NF-κB) and signal transduction and transcriptional activator 3 (STAT3) play important roles in inflammatory responses. Among them, IL-6 is a pleiotropic cytokine that is a major player in chronic inflammation (which is closely associated with chronic inflammatory diseases, autoimmune diseases, and cancer) and cytokine storm (such as the cytokine storm of COVID-19) (41–45). The synergistic action of NF-κB and STAT3 can induce super activation of NF-κB and subsequently produce a variety of inflammatory factors (46). Since IL-6 is a target of NF-κB, both NF-κB and STAT3 are activated in non-immune cells, triggering a positive feedback loop for NF-κB activation via the IL-6 STAT3 axis. This positive feedback loop is called the IL-6 amplifier (IL-6 Amp), which can be further enhanced because activated IL-6 Amp can enhance chemokine production and recruit lymphocytes in lesions, including Th17 cells (46–51). Inflammatory bowel disease, including chronic inflammatory disease, autoimmune diseases and cancer) is a non-immune cells and immune cells through complex interactions between IL - 6 Amp induction, confirmed the model between inflammation and cancer is a process of continuous, rather than by tissue specificity immune tolerance and the destruction of the cancer-causing mutations cause (52). In conclusion, exposure to mixed chemicals (PFASs, PAHs, and Metals), as long-term pro-inflammatory factors in human beings, induces chronic inflammation in human body by triggering IL-6 Amp, which not only affects human health, but also continuously increases medical expenditure (53, 54).

This study is the first to describe the effects of a chemical cocktail on immune inflammatory indicators. However, our study has several limitations. First, due to the cross-sectional design of the study, we were unable to determine a causal relationship between immune-inflammatory markers and chemical levels. Second, abnormal immune-inflammatory indicators are a chronic biochemical process that develops over a long period of time. Chemical exposure levels are determined from blood or urine samples, which represent only recent exposure and may not properly reflect long-term exposure. Third, we have identified only a link between the chemical mix in the general population and immune-inflammatory indicators. More work is needed to further assess the effects of chemical mixtures on immune-inflammatory indicators in specific populations stratified by sex and age (workers, pregnant women, etc.). Fourthly, due to the limited database of genes induced by detected chemicals, some important weighted chemicals in this study have not been retrieved in this database, so there may be some deviations in the results obtained from the analysis.

To our knowledge, this is the first study that environmental chemicals (PFAHs, PAHs, metallic and non-metallic elements) co-exposure measured in human blood or urine with peripheral blood immune-inflammatory indicators. The results showed that PFDE, PFUA, As and Hg had a high negative weight on the immune inflammation spectrum, while P01, P02, P04, and Cd had a high positive weight on the immune inflammation spectrum, and the overall exposure trend was mainly to promote lymphocyte-mediated immune inflammation. This inflammatory response may be related to the activation of IL-6 Amp by environmental chemicals. Although a potential reverse causality cannot be ruled out due to the cross-sectional study design. These results provide important evidence and theoretical basis for exploring the promoting effects of environmental chemical pollution on chronic inflammation and immune- inflammatory diseases (chronic inflammatory diseases, autoimmune diseases and cancer).

## Data availability statement

Publicly available datasets were analyzed in this study. This data can be found at: https://www.cdc.gov/nchs/nhanes/index.htm.

## Ethics statement

The studies involving human participants were reviewed and approved by National Center for Health Statistics (NCHS) Ethics Review Board. Written informed consent to participate in this study was provided by the participants' legal guardian/next of kin.

## Author contributions

YL: writing original draft, data curation, software, and writing review and editing. ZZ: methodology, investigation, and data curation. DH: methodology. YZ: investigation, data curation, and funding acquisition. XY: project administration, writing review and editing, and funding acquisition. SC: project administration, writing review and editing, supervision, and funding acquisition. All authors contributed to the article and approved the submitted version.

## Funding

This work was supported by the Shaanxi Academy of Traditional Chinese Medicine Nursery Cultivation Plan Project (2021-09), National Natural Science Foundation of General Project (82174386), and Shaanxi Provincial Key Industry Innovation Chain (2021ZDLSF04-12 and 2019ZDLSF04-08).

## Conflict of interest

The authors declare that the research was conducted in the absence of any commercial or financial relationships that could be construed as a potential conflict of interest.

## Publisher's note

All claims expressed in this article are solely those of the authors and do not necessarily represent those of their affiliated organizations, or those of the publisher, the editors and the reviewers. Any product that may be evaluated in this article, or claim that may be made by its manufacturer, is not guaranteed or endorsed by the publisher.
