# Supplementary material for: Association between environmental chemicals co-exposure and peripheral blood immune-inflammatory indicators
Source: Front Public Health. 2022 Nov 22;10:980987. doi: 10.3389/fpubh.2022.980987 (PMC9725172; doi:10.3389/fpubh.2022.980987)
Supplement: Supplementary file 1 [file Data_Sheet_1.docx]

Table 1. Distribution according to exposures and immunoinflammatory biomarkers of the study population (n = 1723).

| **Abbreviation** | **Description** | **Units** | **Matrix** | **Detect-ion rate** | **GM** | **AM** | **25th percentile** | **50th percentile** | **75th percentile** |
| --- | --- | --- | --- | --- | --- | --- | --- | --- | --- |
| PFASs | Perfluoroalkyl substances |  |  |  |  |  |  |  |  |
| PFDE | Perfluorodecanoic acid | ng/mL | Blood | 87.5 | 0.21 | 0.31 | 0.13 | 0.2 | 0.32 |
| PFOS | Perfluorooctane sulfonic acid | ng/mL | Blood | 87.5 | 6.28 | 9.21 | 3.815 | 6.4 | 10.8 |
| PFUA | Perfluoroundecanoic acid | ng/mL | Blood | 87.5 | 0.15 | 0.25 | 0.07 | 0.13 | 0.24 |
| PFNA | Perfluorononanoic acid | ng/mL | Blood | 87.5 | 0.92 | 1.19 | 0.61 | 0.89 | 1.36 |
| PFOA | Perfluorooctanoic acid | ng/mL | Blood | 87.5 | 2.00 | 2.46 | 1.41 | 2.06 | 2.91 |
| PFHS | Perfluorohexane sulfonic acid | ng/mL | Blood | 87.5 | 1.20 | 1.85 | 0.7 | 1.22 | 2.11 |
| MPAH | 2-(N-methyl-PFOSA) acetate | ng/mL | Blood | 87.5 | 0.13 | 0.23 | 0.06 | 0.1 | 0.25 |
| PAHs | Polycyclic aromatic hydrocarbons |  |  |  |  |  |  |  |  |
| P01 | 1-hydroxynaphthalene | ng/mL | Urinary | 96.1 | 1780.86 | 18647.00 | 614 | 1468 | 4508 |
| P02 | 2-hydroxynaphthalene | ng/mL | Urinary | 96.1 | 4702.90 | 8925.00 | 2131 | 4851 | 11020 |
| P03 | 3-hydroxyfluorene | ng/mL | Urinary | 95.9 | 98.30 | 267.60 | 40 | 81 | 207.5 |
| P04 | 2-hydroxyfluorene | ng/mL | Urinary | 96.1 | 255.37 | 548.20 | 114 | 229 | 521.5 |
| P05 | 3-hydroxyphenanthrene | ng/mL | Urinary | 96.0 | 66.75 | 118.20 | 32.5 | 65 | 135 |
| P06 | 1-hydroxyphenanthrene | ng/mL | Urinary | 96.1 | 126.23 | 193.40 | 70 | 126 | 235 |
| P07 | 2-hydroxyphenanthrene | ng/mL | Urinary | 96.0 | 65.68 | 103.80 | 35 | 64 | 121 |
| P10 | 1-hydroxypyrene | ng/mL | Urinary | 95.9 | 112.81 | 200.90 | 54 | 112 | 228.5 |
| P17 | 9-hydroxyfluorene | ng/mL | Urinary | 96.0 | 265.72 | 511.50 | 123.5 | 257 | 564 |
| P19 | 4-phenanthrene | ng/mL | Urinary | 95.9 | 21.86 | 34.34 | 11 | 21 | 39 |
| Metallic and nonmetallic elements |  |  |  |  |  |  |  |  |  |
| As | Arsenic | ug/L | Urinary | 96.5 | 8.22 | 19.00 | 3.71 | 7.39 | 16.73 |
| Cd | Cadmium | ug/L | Blood | 88.4 | 0.31 | 0.47 | 0.17 | 0.28 | 0.51 |
| Pb | Lead | ug/L | Blood | 88.4 | 1.00 | 1.34 | 0.6 | 0.96 | 1.56 |
| Hg | Lead | ug/L | Blood | 88.4 | 0.81 | 1.55 | 0.38 | 0.74 | 1.62 |
| Inflammatory immune biomarkers |  |  |  |  |  |  |  |  |  |
| Wbc | White blood cell | 1000 cell/uL | Blood | 88.7 | 6.58 | 6.86 | 5.50 | 6.60 | 8.00 |
| Neu | Neutrophil | 1000 cell/uL | Blood | 88.7 | 3.71 | 4.02 | 2.80 | 3.80 | 4.80 |
| Lym | Lymphocyte | 1000 cell/uL | Blood | 88.7 | 1.99 | 2.09 | 1.60 | 2.00 | 2.50 |
| NLR | Neutrophil-to-lymphocyte ratio | —— | —— | —— | 1.86 | 2.10 | 1.36 | 1.85 | 2.54 |

Table 2 Association between single environmental chemical exposure and immunoinflammatory biomarkers based on multivariate linear regression (n=1723).

| **Categories** | **Outcome** | **Continuous** | **Q2** | **Q3** | **Q4** | **P for trend** |
| --- | --- | --- | --- | --- | --- | --- |
|  |  | **β(95%CI)** | **β(95%CI)** | **β(95%CI)** | **β(95%CI)** |  |
| PFASs |  |  |  |  |  |  |
| PFDE | Wbc | -0.14(-0.22,-0.06) | -0.23(-0.49,0.02) | -0.22(-0.49,0.04) | -0.50(-0.77,-0.23) | **0.001** |
|  | Neu | -0.14(-0.21,-0.07) | -0.24(-0.46,-0.03) | -0.29(-0.51,-0.07) | -0.48(-0.70,-0.25) | **<0.001** |
|  | Lym | 0.01(-0.02,0.04) | 0.04(-0.05,0.12) | 0.07(-0.01,0.16) | 0.02(-0.06,0.11) | 0.422 |
|  | NLR | -0.11(-0.16,-0.06) | -0.23(-0.38,-0.08) | -0.31(-0.46,-0.16) | -0.35(-0.51,-0.19) | **<0.001** |
| PFOS | Wbc | -0.10(-0.19,-0.01) | -0.05(-0.31,0.21) | -0.20(-0.47,0.06) | -0.31(-0.59,-0.03) | **0.022** |
|  | Neu | -0.10(-0.17,-0.03) | -0.07(-0.29,0.14) | -0.27(-0.49,-0.05) | -0.29(-0.52,-0.05) | **0.007** |
|  | Lym | 0.00(-0.03,0.03) | 0.03(-0.06,0.11) | 0.06(-0.03,0.14) | -0.02(-0.11,0.08) | 0.964 |
|  | NLR | -0.05(-0.10,0.00) | -0.08(-0.23,0.08) | -0.19(-0.34,-0.03) | -0.13(-0.29,0.04) | 0.066 |
| PFUA | Wbc | -0.14(-0.21,-0.06) | -0.33(-0.65,-0.02) | -0.20(-0.44,0.04) | -0.49(-0.74,-0.24) | **<0.001** |
|  | Neu | -0.12(-0.19,-0.05) | -0.27(-0.53,-0.01) | -0.19(-0.39,0.02) | -0.42(-0.64,-0.21) | **<0.001** |
|  | Lym | -0.01(-0.03,0.02) | -0.04(-0.14,0.07) | 0.01(-0.07,0.09) | -0.04(-0.12,0.05) | 0.615 |
|  | NLR | -0.07(-0.12,-0.03) | -0.18(-0.37,0.00) | -0.14(-0.28,0.00) | -0.25(-0.39,-0.10) | **0.002** |
| PFNA | Wbc | -0.05(-0.13, 0.04) | 0.04(-0.22,0.30) | -0.01(-0.26,0.25) | -0.16(-0.42,0.11) | 0.266 |
|  | Neu | -0.06(-0.13,0.01) | -0.04(-0.26,0.18) | -0.02(-0.24,0.19) | -0.21(-0.43,0.01) | 0.104 |
|  | Lym | 0.02(-0.01,0.05) | 0.09(0.01,0.18) | 0.04(-0.05,0.12) | 0.08(-0.01,0.17) | 0.165 |
|  | NLR | -0.07(-0.12,-0.02) | -0.16(-0.31,-0.01) | -0.14(-0.29,0.01) | -0.25(-0.40,-0.09) | **0.005** |
| PFOA | Wbc | -0.02(-0.11, 0.06) | 0.12(-0.13,0.38) | 0.04(-0.22,0.30) | -0.09(-0.36,0.17) | 0.578 |
|  | Neu | -0.04(-0.11,0.03) | 0.02(-0.19,0.24) | -0.04(-0.26,0.18) | -0.13(-0.36,0.09) | 0.287 |
|  | Lym | 0.01(-0.01,0.04) | 0.07(-0.02,0.15) | 0.07(-0.01,0.16) | 0.04(-0.05,0.13) | 0.312 |
|  | NLR | -0.03(-0.08,0.02) | -0.02(-0.17,0.13) | -0.02(-0.17,0.13) | -0.10(-0.25,0.06) | 0.270 |
| PFHS | Wbc | -0.02(-0.11, 0.06) | -0.12(-0.38,0.13) | -0.13(-0.39,0.13) | -0.11(-0.37,0.15) | 0.558 |
|  | Neu | -0.04(-0.11,0.03) | -0.19(-0.40,0.03) | -0.14(-0.36,0.08) | -0.17(-0.39,0.05) | 0.240 |
|  | Lym | 0.00(-0.02,0.03) | 0.08(-0.01,0.16) | 0.00(-0.08,0.09) | 0.03(-0.05,0.12) | 0.725 |
|  | NLR | -0.03(-0.08,0.01) | -0.19(-0.34,-0.04) | -0.10(-0.25,0.05) | -0.14(-0.30,0.01) | 0.174 |
| MPAH | Wbc | -0.02(-0.10, 0.05) | -0.38(-0.75,-0.01) | -0.15(-0.39,0.08) | -0.05(-0.28,0.19) | 0.515 |
|  | Neu | 0.00(-0.06,0.06) | -0.27(-0.58,0.04) | -0.11(-0.30,0.09) | 0.02(-0.18,0.21) | 0.918 |
|  | Lym | -0.02(-0.04,0.01) | -0.07(-0.20,0.05) | -0.02(-0.09,0.06) | -0.06(-0.13,0.02) | 0.175 |
|  | NLR | 0.02(-0.03,0.06) | 0.00(-0.22,0.21) | -0.01(-0.15,0.12) | 0.07(-0.07,0.20) | 0.424 |
| PAHs |  |  |  |  |  |  |
| P01 | Wbc | 0.25(0.17, 0.34) | -0.09(-0.34,0.16) | -0.05(-0.30,0.20) | 0.80(0.54,1.05) | **<0.001** |
|  | Neu | 0.20( 0.13,0.27) | -0.02(-0.23,0.19) | 0.01(-0.20,0.22) | 0.62(0.41,0.84) | **<0.001** |
|  | Lym | 0.04(0.02, 0.07) | -0.08(-0.16,0.01) | -0.06(-0.15,0.02) | 0.13(0.04,0.21) | **0.002** |
|  | NLR | 0.06( 0.01,0.11) | 0.07(-0.08,0.22) | 0.08(-0.07,0.23) | 0.19(0.04,0.34) | **0.017** |
| P02 | Wbc | 0.33(0.24, 0.41) | 0.15(-0.11,0.40) | 0.26(0.00,0.51) | 1.09(0.83,1.36) | **<0.001** |
|  | Neu | 0.27( 0.20,0.34) | 0.18(-0.03,0.39) | 0.26(0.04,0.47) | 0.92(0.70,1.14) | **<0.001** |
|  | Lym | 0.04( 0.01,0.07) | -0.06(-0.15,0.02) | -0.01(-0.09,0.08) | 0.11(0.02,0.20) | **0.008** |
|  | NLR | 0.08( 0.03,0.13) | 0.15(0.00,0.30) | 0.10(-0.05,0.25) | 0.30(0.14,0.46) | **0.002** |
| P03 | Wbc | 0.28(0.20, 0.37) | -0.20(-0.45,0.06) | -0.05(-0.30,0.21) | 0.86(0.60,1.12) | **<0.001** |
|  | Neu | 0.23( 0.16,0.30) | -0.12(-0.34,0.09) | 0.07(-0.15,0.28) | 0.68(0.46,0.90) | **<0.001** |
|  | Lym | 0.04( 0.01,0.07) | -0.05(-0.13,0.04) | -0.10(-0.19,-0.01) | 0.13(0.05,0.22) | **0.008** |
|  | NLR | 0.06( 0.01,0.11) | -0.08(-0.23,0.07) | 0.07(-0.08,0.23) | 0.15(0.00,0.31) | **0.013** |
| P04 | Wbc | 0.29(0.21, 0.37) | -0.16(-0.41,0.10) | 0.20(-0.06,0.45) | 0.84(0.58,1.10) | **<0.001** |
|  | Neu | 0.24( 0.17,0.31) | -0.07(-0.28,0.15) | 0.22(0.01,0.44) | 0.69(0.48,0.91) | **<0.001** |
|  | Lym | 0.04( 0.01,0.06) | -0.06(-0.15,0.02) | -0.03(-0.12,0.05) | 0.11(0.02,0.19) | **0.009** |
|  | NLR | 0.07( 0.02,0.12) | -0.03(-0.18,0.11) | 0.09(-0.06,0.24) | 0.19(0.04,0.34) | **0.005** |
| P05 | Wbc | 0.22(0.14, 0.30) | 0.03(-0.22,0.29) | 0.16(-0.09,0.42) | 0.68(0.42,0.94) | **<0.001** |
|  | Neu | 0.18( 0.11,0.25) | 0.03(-0.19,0.24) | 0.14(-0.07,0.36) | 0.56(0.34,0.77) | **<0.001** |
|  | Lym | 0.03( 0.00,0.06) | 0.00(-0.08,0.09) | 0.02(-0.06,0.11) | 0.08(0.00,0.17) | **0.039** |
|  | NLR | 0.04(-0.01,0.09) | -0.04(-0.18,0.11) | -0.02(-0.17,0.13) | 0.13(-0.02,0.28) | 0.097 |
| P06 | Wbc | 0.11(0.03, 0.19) | 0.02(-0.23,0.28) | 0.16(-0.10,0.41) | 0.34(0.08,0.60) | **0.006** |
|  | Neu | 0.11( 0.04,0.17) | 0.06(-0.15,0.28) | 0.18(-0.03,0.40) | 0.32(0.10,0.54) | **0.002** |
|  | Lym | 0.01(-0.02,0.03) | -0.04(-0.12,0.05) | -0.03(-0.11,0.06) | 0.01(-0.07,0.10) | 0.681 |
|  | NLR | 0.02(-0.03,0.07) | 0.02(-0.13,0.17) | 0.03(-0.12,0.18) | 0.07(-0.08,0.22) | 0.383 |
| P07 | Wbc | 0.20( 0.12, 0.29) | 0.09(-0.17,0.35) | 0.28(0.02,0.54) | 0.66(0.40,0.92) | **<0.001** |
|  | Neu | 0.17( 0.10,0.23) | 0.12(-0.09,0.34) | 0.24(0.03,0.46) | 0.55(0.33,0.77) | **<0.001** |
|  | Lym | 0.02( 0.00,0.05) | -0.04(-0.13,0.04) | 0.01(-0.07,0.10) | 0.07(-0.01,0.16) | 0.074 |
|  | NLR | 0.04(-0.01,0.09) | 0.01(-0.14,0.16) | 0.04(-0.11,0.19) | 0.13(-0.02,0.29) | 0.112 |
| P10 | Wbc | 0.18(0.10, 0.26) | -0.11(-0.37,0.15) | 0.23(-0.02,0.49) | 0.46(0.20,0.73) | **<0.001** |
|  | Neu | 0.16( 0.09,0.22) | -0.09(-0.31,0.12) | 0.22(0.00,0.43) | 0.40(0.18,0.63) | **<0.001** |
|  | Lym | 0.02(-0.01,0.04) | -0.01(-0.10,0.07) | 0.03(-0.06,0.11) | 0.04(-0.05,0.12) | 0.226 |
|  | NLR | 0.04(-0.01,0.09) | -0.13(-0.28,0.02) | 0.05(-0.10,0.20) | 0.08(-0.08,0.23) | 0.101 |
| P17 | Wbc | 0.24(0.16, 0.32) | 0.16(-0.09,0.42) | 0.39(0.13,0.65) | 0.75(0.49,1.01) | **<0.001** |
|  | Neu | 0.21( 0.14,0.28) | 0.22(0.00,0.43) | 0.37(0.15,0.59) | 0.67(0.45,0.89) | **<0.001** |
|  | Lym | 0.02(-0.01,0.05) | -0.08(-0.16,0.01) | 0.00(-0.09,0.08) | 0.04(-0.05,0.13) | 0.172 |
|  | NLR | 0.07( 0.03,0.12) | 0.16(0.01,0.31) | 0.16(0.01,0.31) | 0.26(0.10,0.41) | **0.003** |
| P19 | Wbc | 0.20(0.12, 0.29) | -0.02(-0.27,0.24) | 0.16(-0.10,0.42) | 0.67(0.41,0.93) | **<0.001** |
|  | Neu | 0.17( 0.10,0.24) | 0.04(-0.17,0.26) | 0.16(-0.06,0.37) | 0.57(0.35,0.79) | **<0.001** |
|  | Lym | 0.03( 0.00,0.05) | -0.07(-0.15,0.02) | -0.01(-0.09,0.08) | 0.07(-0.02,0.16) | 0.062 |
|  | NLR | 0.04(-0.01,0.09) | 0.02(-0.13,0.17) | 0.02(-0.13,0.17) | 0.16(0.00,0.31) | 0.086 |
| Metallic and nonmetallic elements |  |  |  |  |  |  |
| As | Wbc | -0.12(-0.20,-0.04) | -0.11(-0.36,0.15) | -0.10(-0.36,0.16) | -0.41(-0.68,-0.14) | **0.005** |
|  | Neu | -0.07(-0.14,0.00) | -0.10(-0.32,0.11) | -0.03(-0.25,0.19) | -0.25(-0.48,-0.03) | 0.064 |
|  | Lym | -0.04(-0.07,-0.02) | 0.00(-0.09,0.08) | -0.07(-0.16,0.02) | -0.13(-0.22,-0.04) | **0.002** |
|  | NLR | -0.01(-0.06,0.04) | -0.08(-0.23,0.07) | 0.06(-0.10,0.21) | -0.07(-0.23,0.08) | 0.765 |
| Cd | Wbc | 0.27(0.18, 0.36) | -0.04(-0.30,0.22) | 0.18(-0.08,0.45) | 0.78(0.50,1.06) | **<0.001** |
|  | Neu | 0.20(0.12, 0.27) | -0.02(-0.24,0.20) | 0.16(-0.07,0.39) | 0.55(0.32,0.79) | **<0.001** |
|  | Lym | 0.06(0.03, 0.09) | 0.02(-0.07,0.10) | 0.03(-0.06,0.12) | 0.19(0.10,0.28) | **<0.001** |
|  | NLR | 0.03(-0.02,0.08) | -0.07(-0.22,0.09) | 0.03(-0.13,0.19) | 0.05(-0.12,0.21) | 0.298 |
| Pb | Wbc | 0.05(-0.05, 0.14) | -0.15(-0.42,0.11) | -0.15(-0.43,0.14) | 0.06(-0.24,0.37) | 0.328 |
|  | Neu | 0.03(-0.05,0.11) | -0.23(-0.45,0.00) | -0.17(-0.41,0.06) | 0.00(-0.25,0.26) | 0.494 |
|  | Lym | 0.00(-0.03,0.03) | 0.03(-0.05,0.12) | 0.01(-0.09,0.10) | 0.00(-0.10,0.10) | 0.902 |
|  | NLR | 0.00(-0.06,0.05) | -0.15(-0.30,0.00) | -0.09(-0.26,0.07) | -0.05(-0.23,0.12) | 0.889 |
| Hg | Wbc | -0.09(-0.18, 0.00) | -0.02(-0.28,0.24) | -0.04(-0.31,0.22) | -0.31(-0.60,-0.03) | 0.039 |
|  | Neu | -0.09(-0.17,-0.02) | -0.09(-0.31,0.12) | -0.13(-0.35,0.09) | -0.29(-0.53,-0.06) | **0.018** |
|  | Lym | 0.00(-0.03,0.03) | 0.08(-0.01,0.16) | 0.10(0.01,0.19) | 0.00(-0.09,0.09) | 0.854 |
|  | NLR | -0.08(-0.14,-0.03) | -0.17(-0.32,-0.02) | -0.24(-0.39,-0.09) | -0.25(-0.42,-0.09) | **0.002** |

Quartile 1 was set as the reference.

Table 3. Association between environmental chemical exposure and immunoinflammatory biomarkers based on weighted quantile sum regression (WQSR) analysis (n=1723).

| **Outcomes** | **Components** | **Positive** | | **Negative** | |
| --- | --- | --- | --- | --- | --- |
|  |  | **βWQS(95% CI)** | **P-value** | **βWQS(95% CI)** | **P-value** |
| Wbc | Total | 0.670(0.387,0.951) | **<0.001** | -0.401( -0.633,-0.168) | **0.001** |
|  | PFASs | -0.174(-0.399,0.167) | 0.157 | -0.499(-0.730,-0.268) | **<0.001** |
|  | PAHs | 0.430(0.249,0.611) | **<0.001** | NA | NA |
|  | Metallic and nonmetallic elements | 0.330(0.148,0.512) | **<0.001** | -0.246(-0.419,-0.073) | **0.006** |
| Neu | Total | 0.454(0.213,0.693) | **<0.001** | NA | NA |
|  | PFAS | -0.156(-0.313,0.173) | 0.177 | -0.453(-0.639,-0.268) | **<0.001** |
|  | PAHs | 0.292(0.139,0.445) | **<0.001** | NA | NA |
|  | Metallic and nonmetallic elements | 0.209(0.0587,0.360) | **<0.001** | -0.181(-0.321,-0.0412) | **0.011** |
| Lym | Total | 0.196(0.096,0.298) | **<0.001** | -0.002(-0.096,0.104) | 0.969 |
|  | PFASs | 0.026(-0.046,0.101) | 0.487 | 0.014(-0.091,0.122) | 0.799 |
|  | PAHs | 0.104(0.046,0.160) | **0.001** | 0.075(0.023,0.125) | **0.004** |
|  | Metallic and nonmetallic elements | 0.098(0.034,0.163) | **0.003** | -0.074(-0.131,-0.016) | **0.012** |
| NLR | Total | -0.033(-0.232,0.166) | 0.744 | -0.334(-0.491,-0.178) | **<0.001** |
|  | PFASs | -0.085(-0.250,0.080) | 0.312 | -0.272(-0.396,-0.149) | **<0.001** |
|  | PAHs | 0.007(-0.103,0.117) | 0.898 | -0.008(-0.103,0.086) | 0.864 |
|  | Metallic and nonmetallic elements | -0.022(-0.153,0.109) | 0.738 | -0.143(-0.245,-0.040) | **0.007** |

*β*: the overall mixture effect from WQS regression index.

Table 4. Posterior inclusion probabilities (PIPs) for chemicals into immunoinflammatory biomarkers using the Bayesian kernel machine regression (BKMR) model (n = 1723).

| **Chemicals** | **Posterior inclusion probabilities** | | | |
| --- | --- | --- | --- | --- |
|  | **Wbc** | **Neu** | **Lym** | **NLR** |
| **Total** |  |  |  |  |
| PFDE | 0.4624 | 0.5576 | 0.1836 | 0.5540 |
| PFOS | 0.3444 | 0.3856 | 0.0448 | 0.3100 |
| PFUA | 0.8132 | 0.9756 | 0.1856 | 0.6892 |
| PFNA | 0.3648 | 0.3828 | 0.1952 | 0.2624 |
| PFOA | 0.3580 | 0.5492 | 0.2568 | 0.2568 |
| PFHS | 0.3000 | 0.3920 | 0.7868 | 0.2156 |
| MPAH | 0.1820 | 0.2500 | 0.0548 | 0.2100 |
| P01 | 0.5876 | 0.5216 | 0.0652 | 0.3552 |
| P02 | 0.3024 | 0.5636 | 0.0316 | 0.2132 |
| P03 | 0.6724 | 0.6376 | 0.2232 | 0.0916 |
| P04 | 0.7856 | 0.8272 | 0.2292 | 0.2124 |
| P05 | 0.3036 | 0.4036 | 0.0636 | 0.2220 |
| P06 | 0.3832 | 0.3440 | 0.0340 | 0.2388 |
| P07 | 0.3884 | 0.4744 | 0.0460 | 0.1444 |
| P10 | 0.1400 | 0.3584 | 0.0608 | 0.1880 |
| P17 | 0.3356 | 0.4700 | 0.0256 | 0.3176 |
| P19 | 0.2712 | 0.5368 | 0.1232 | 0.1936 |
| **As** | 0.7060 | 0.6816 | 0.2580 | 0.2208 |
| Cd | 0.2428 | 0.3212 | 0.0460 | 0.1556 |
| Pb | 0.2508 | 0.5084 | 0.1032 | 0.1516 |
| Hg | 0.3568 | 0.3648 | 0.0644 | 0.3956 |
| **PFASs** |  |  |  |  |
| PFDE | 0.0220 | 0.6952 | 0.1376 | 0.6952 |
| PFOS | 0.0024 | 0.4812 | 0.0408 | 0.4812 |
| PFUA | 1.0000 | 0.8392 | 0.2320 | 0.8392 |
| PFNA | 0.0068 | 0.6528 | 0.3500 | 0.6528 |
| PFOA | 0.0400 | 0.5420 | 0.0908 | 0.5420 |
| PFHS | 0.0000 | 0.3620 | 0.5260 | 0.3620 |
| MPAH | 0.0000 | 0.4804 | 0.0256 | 0.4804 |
| **PAHs** |  |  |  |  |
| P01 | 0.9512 | 0.8500 | 0.5992 | 0.0440 |
| P02 | 0.0740 | 0.2980 | 0.0000 | 0.0684 |
| P03 | 1.0000 | 1.0000 | 0.5628 | 0.0788 |
| P04 | 0.9244 | 0.9348 | 0.4696 | 0.1044 |
| P05 | 0.9844 | 0.9168 | 0.6424 | 0.0584 |
| P06 | 0.2932 | 0.4544 | 0.0248 | 0.0568 |
| P07 | 0.9576 | 0.8748 | 0.1364 | 0.0288 |
| P10 | 0.0380 | 0.0548 | 0.1544 | 0.0216 |
| P17 | 0.0056 | 0.0464 | 0.0676 | 0.0528 |
| P19 | 0.0916 | 0.2640 | 0.0388 | 0.0212 |
| **Metallic and nonmetallic elements** |  |  |  |  |
| As | 1.0000 | 1.0000 | 0.5648 | 0.0056 |
| Cd | 1.0000 | 1.0000 | 1.0000 | 0.1600 |
| Pb | 0.0164 | 0.0888 | 0.0240 | 0.0244 |
| Hg | 0.0084 | 0.0696 | 0.1820 | 0.9620 |

Table 5. Genes associated with the immunoinflammatory biomarkers and high-weight chemicals.

| Chemical Name | Chemical ID | Disease Name | Disease ID | Inference Network |
| --- | --- | --- | --- | --- |
| PFUA | C586085 | Inflammation | MESH:D007249 | AGT\|AKT1\|BDNF\|CCK\|CCL4\|CRHR2\|CXCL8\|EDN1\|FGF2\|HMOX1\|IL6\|  PPARA\|PPARG\|SOD1\|TF\|VEGFA |
| PFDE | C036567 | Inflammation | MESH:D007249 | AKT1\|CYP3A23-3A1\|HMOX1\|IL10\|IL6\|NOS2\|PPARA\|PPARG\|TNF\|VEGFA |
| PFHS | C471071 | Inflammation | MESH:D007249 | ABCB4\|ADIPOQ\|APOA1\|BDNF\|CD3E\|CYP2B1\|CYP3A23-3A1\|  HMOX1\|LEP\|NOS2\|NR1H4\|PPARA\|PPARG\|SLC22A5\|TNFSF15 |
| PFOS | Not available | Not available | Not available | Not available |
| P01 | C029350 | Inflammation | MESH:D007249 | AHR\|PPARA\|TRPA1 |
| P02 | C028405 | Inflammation | MESH:D007249 | AHR |
| P04 | [C477779](https://meshb.nlm.nih.gov/record/ui?ui=C477779) | Not available | Not available | Not available |
| Ca | D002104 | Inflammation | MESH:D007249 | AGT\|AHR\|AKT1\|APOA1\|ATP7B\|BDNF\|CALCA\|CASP1\|CCL2\|COL2A1\|CRP\|  CSF2\|CXCL2\|CXCL8\|DUSP10\|EDN1\|EFNB1\|EGR1\|EIF4EBP1\|EPO\|FGF2\|  GAL\|HMGB1\|HMOX1\|ICAM1\|IFNG\|IKBKB\|IL10\|IL13\|IL17A\|IL1A\|IL1B\|  IL1RN\|IL6\|JAK2\|KYNU\|LCN2\|LTF\|MIF\|MIR21\|MIR22\|MMP2\|MMP9\|MPO\|  NGF\|NLRP3\|NOS2\|PARP1\|PLAUR\|PPARA\|PPARG\|PTGER4\|PTGS2\|SCGB1A1\|  SOD1\|STAT3\|SULT2B1\|TF\|TFRC\|TGFA\|TGFB1\|TIMP1\|TLR4\|TNF\|TNFAIP3\|  TNFRSF11B\|TNFSF15\|UCN\|VEGFA\|WDR1\|ZFP36 |
| As | D001151 | Inflammation | MESH:D007249 | ADIPOQ\|AGER\|AHR\|AKT1\|ANGPT1\|ASIC2\|ASIC3\|BDNF\|CASP1\|CCL2\|  CCL3\|CCL4\|CD3E\|CHRNA4\|CRHR2\|CRP\|CSF2\|CXCL2\|CXCL8\|CXCR3\|  DUSP10\|EFNB1\|EGR1\|EIF4EBP1\|F2R\|FGF2\|GATA3\|HMOX1\|ICAM1\|  IFNG\|IKBKB\|IL10\|IL13\|IL17A\|IL1A\|IL1B\|IL1RN\|IL6\|IRF7\|JAK2\|LEP\|LTF\|  MBP\|MIF\|MIR21\|MMP2\|MMP9\|MPO\|MYD88\|NGF\|NLRP3\|NOS2\|PARP1\|  PLAUR\|PPARA\|PPARG\|PROCR\|PTGS2\|RORC\|SCGB1A1\|SLC22A5\|SOD1\|  STAT3\|SULT2B1\|TBX21\|TFRC\|TGFA\|TGFB1\|TLR4\|TNF\|TNFAIP3\|TNFSF15\|  TP73\|TSC2\|TSLP\|UCN3\|VEGFA |


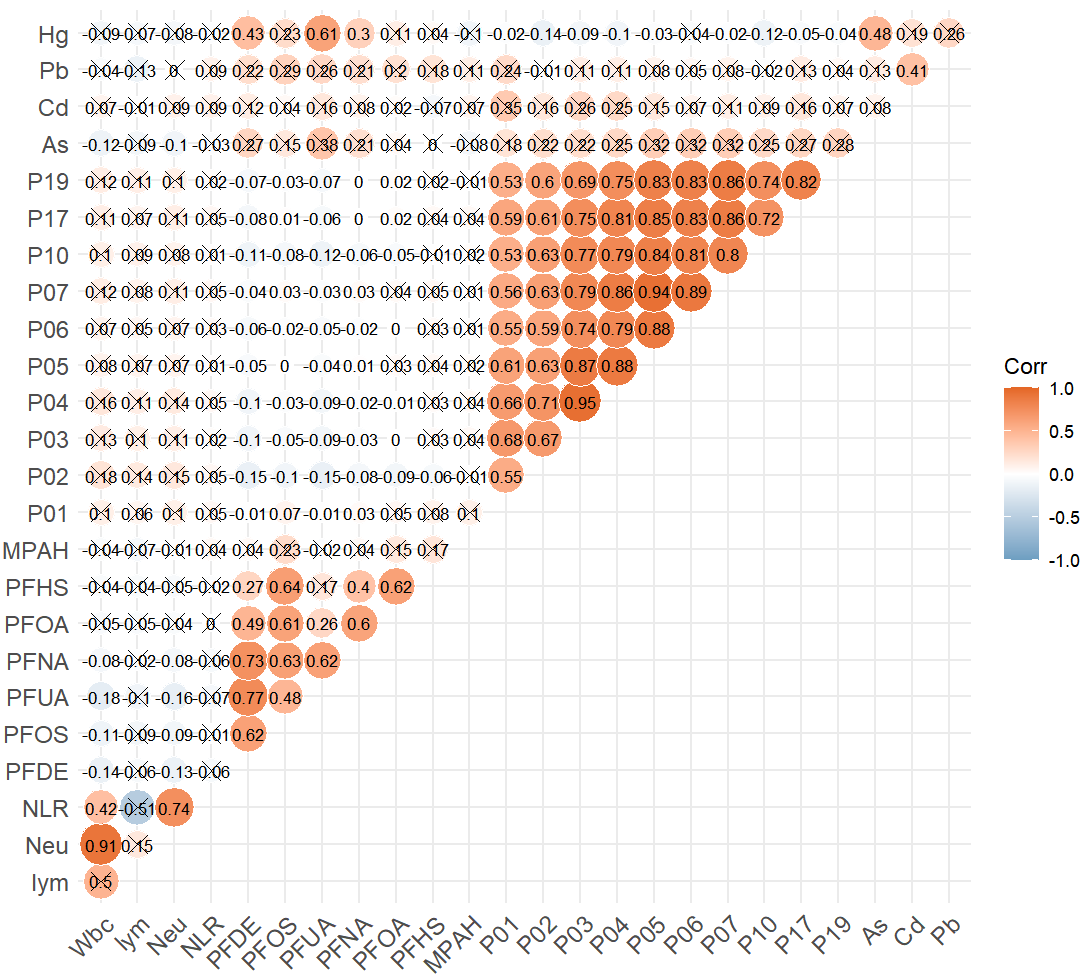


**FIGURE 1** | Pearson correlation matrix of 21 chemicals and 4 immune inflammation indicators in the population (n = 1723). Cross shape means the correlations were not statistically significant (P > 0.01).


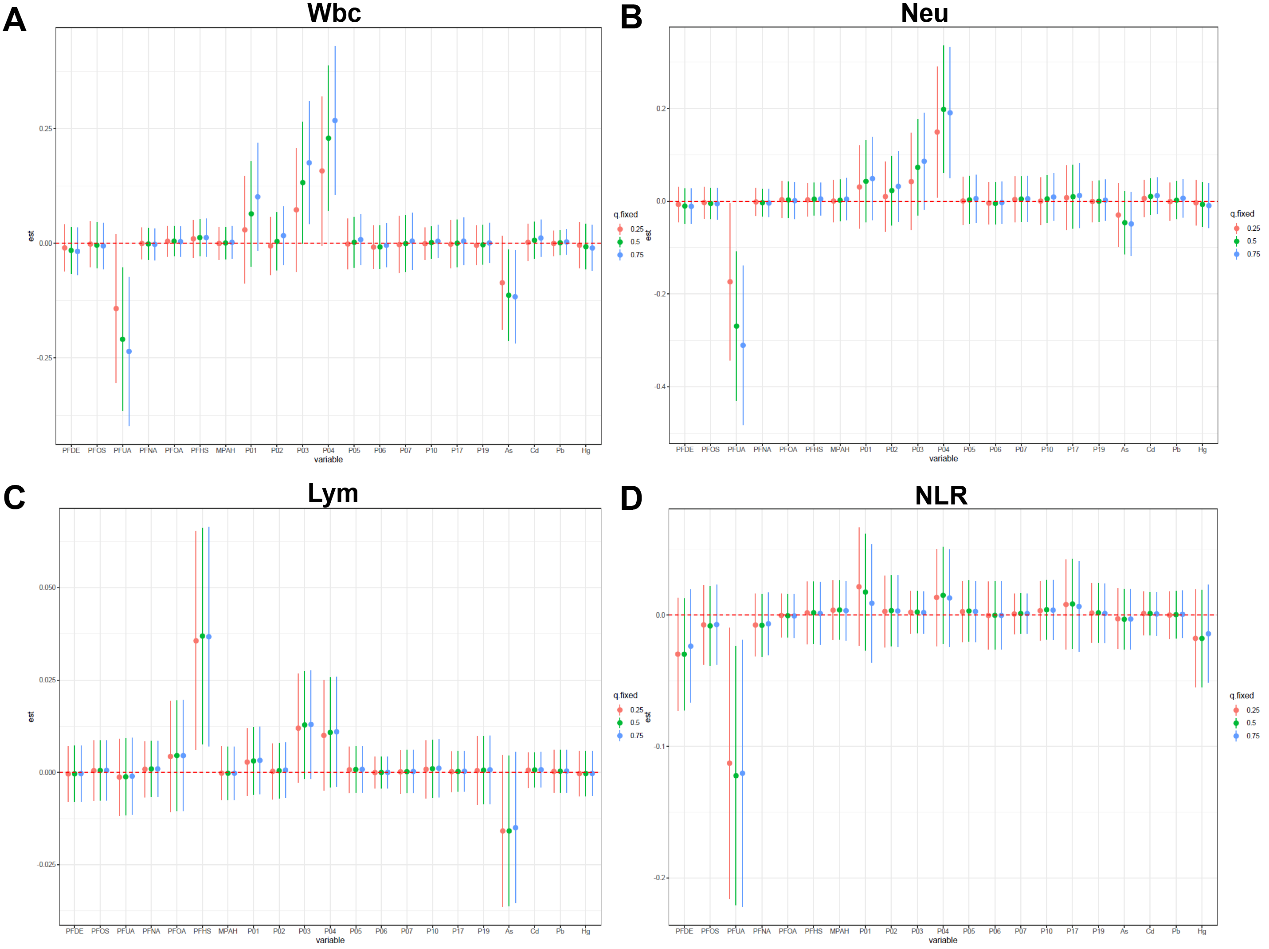


**FIGURE 2 |** Associations of Wbc (A), Neu (B), Lym (C), and NLR (D) with chemical levels were estimated by Bayesian Kernel Machine Regression (BKMR) in total population, when chemical levels were held at their corresponding 25th (red), 50th (green) or 75th (blue) percentile, respectively. Models (total) were adjusted for gender, age, BMI, educational levels, income.


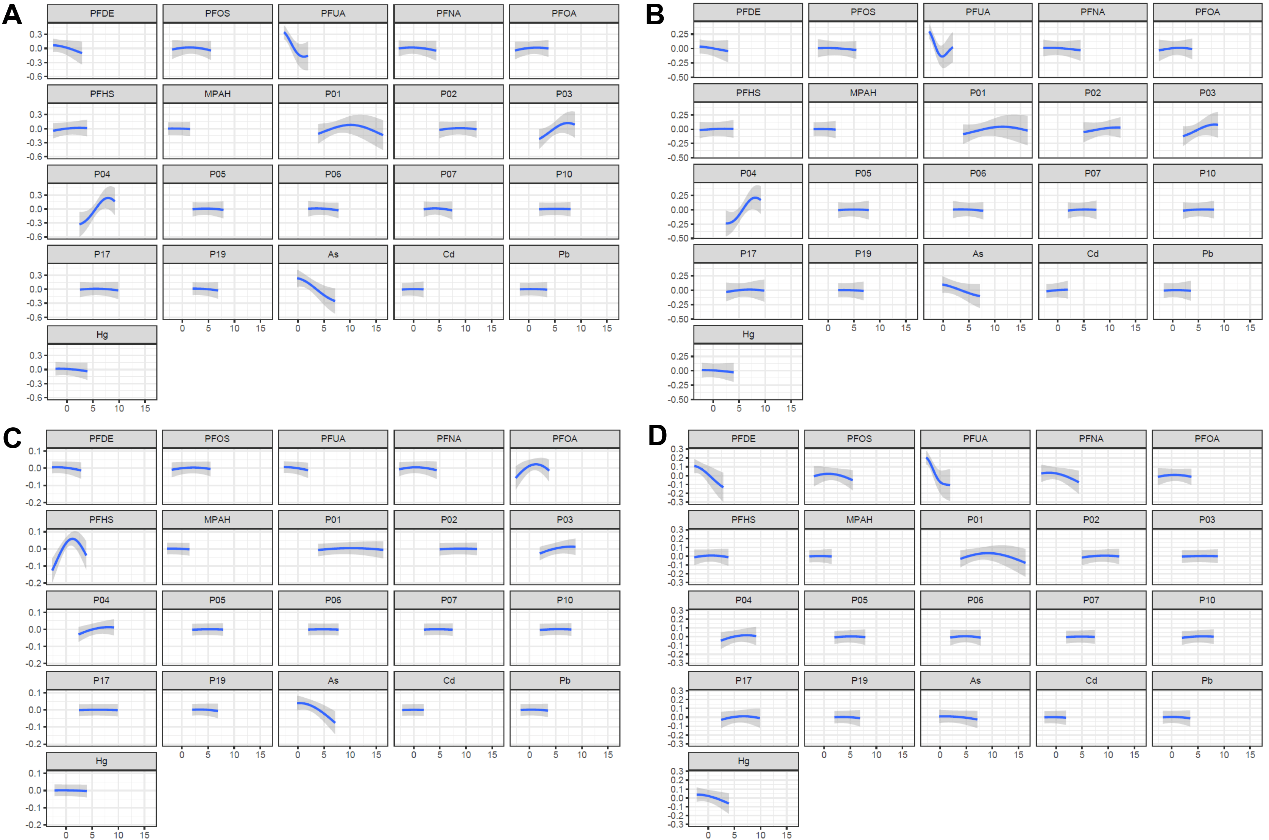


**FIGURE 3 |** Univariate exposure response function (95% CI) between chemical levels and Wbc (A), Neu (B), Lym (C), and NLR (D) while fixing the levels of other chemicals at median values. The results were assessed by the BKMR model adjusted for gender, age, BMI, educational levels, income.
